# HMGA1 Has Predictive Value in Response to Chemotherapy in Gastric Cancer

**DOI:** 10.3390/curroncol29010005

**Published:** 2021-12-23

**Authors:** Diana Pádua, Débora Filipa Pinto, Paula Figueira, Carlos Filipe Pereira, Raquel Almeida, Patrícia Mesquita

**Affiliations:** 1 i3S—Institute for Research and Innovation in Health, University of Porto, 4200-135 Porto, Portugal; dpadua@ipatimup.pt (D.P.); deborafilipapinto@gmail.com (D.F.P.); paulafigueiracp@gmail.com (P.F.); ralmeida@ipatimup.pt (R.A.); 2 IPATIMUP—Institute of Molecular Pathology and Immunology, University of Porto, 4200-465 Porto, Portugal; 3 ICBAS—Institute of Biomedical Sciences Abel Salazar, University of Porto, 4050-313 Porto, Portugal; 4 CNC—Center for Neuroscience and Cell Biology, University of Coimbra, 3004-517 Coimbra, Portugal; filipe.pereira@cnc.uc.pt; 5 Cell Reprogramming in Hematopoiesis and Immunity Laboratory, Molecular Medicine and Gene Therapy, Lund Stem Cell Center, Lund University, BMC A12, 221 84 Lund, Sweden; 6 Wallenberg Center for Molecular Medicine, Lund University, 221 84 Lund, Sweden; 7 Faculty of Medicine, University of Porto, 4200-319 Porto, Portugal; 8 Biology Department, Faculty of Sciences, University of Porto, 4169-007 Porto, Portugal

**Keywords:** HMGA1, gastric cancer, prognosis, predictive value, chemotherapy

## Abstract

Gastric cancer is a serious health problem worldwide. Although its incidence is decreasing, the five-year survival rate remains low. Thus, it is essential to identify new biomarkers that could promote better diagnosis and treatment of patients with gastric cancer. High-mobility group AT-hook 1 (HMGA1) is a non-histone, chromatin-binding protein that has been found overexpressed in several tumor types. It has been correlated with invasion, metastasis, and drug resistance, leading to worse patient survival. The aim of this work was to evaluate the clinical value of HMGA1 in gastric cancer. HMGA1 expression was analyzed by immunohistochemistry in a single hospital series (*n* = 323) of gastric adenocarcinoma cases (stages I to IV) with clinicopathological and treatment data. In this series, HMGA1 expression showed no significant relevance as a prognostic biomarker. Nevertheless, a significantly better overall survival was observed in cases with high levels of HMGA1 when they were treated with chemotherapy, compared to the nontreated ones, implying that they can benefit more from treatment than patients with low expression of HMGA1. We thereby show for the first time that HMGA1 expression has a substantial value as a biomarker of response to chemotherapy in gastric cancer.

## 1. Introduction

Gastric cancer (GC) remains one of the most commonly diagnosed cancers and the fourth leading cause of global cancer mortality [1]. The majority of GCs are adenocarcinoma (90% to 95%). According to the Laurén classification system, the gastric adenocarcinomas can be histologically classified as intestinal, diffuse and mixed type, depending on tissue architecture and glandular patterns. The diffuse-type involves non-cohesive and poorly differentiated tumors, with no gland formation, while intestinal-type tumors are moderate to differentiated tumors, having glandular structures. The mixed type presents features from both the diffuse and intestinal types [2,3]. GC treatment options depend on the stage of the disease at the time of diagnosis. At the early stages, surgical resection of the tumor is the preferred treatment [4]. For stages IB–III, surgery is recommended along with perioperative or adjuvant chemotherapy in order to improve overall patient survival due to the probable relapse after resection [4]. Yet, a significant number of patients have advanced disease at diagnosis, and, consequently, surgery is no longer an option [5]. Therefore, for stage IV, first-line chemotherapy with doublet or triplet platinum/fluoropyrimidine combinations or second-line chemotherapy using taxane, ramucirumab or irinotecan alone or in combination with paclitaxel are recommended [4]. Despite all the improvements registered in the diagnosis and treatment of GC in the last decades, patient prognosis remains poor [5,6]. In Europe, five-year survival rates are enormously diverse between different countries, with a maximum value of 37.5% obtained when best practices are used [7]. The heterogeneity of GC at the molecular level plays an important role in terms of tumor aggressiveness, regardless of tumor extension or tumor type [8,9]. This reveals an urgent need to better understand the disease in order to improve diagnostic techniques and treatments. The validation of new biomarkers can allow for a more personalized practice by making it possible to distinguish between patients who may benefit more from systemic treatment at a specific stage of the disease [10,11]. These biomarkers should comprise transcription factors (TFs), as they are essential in the control of regulatory gene networks and cell fate, and their normal activity is usually altered in different types of cancer [11,12,13].

The transcription factor high-mobility group AT-hook 1 (HMGA1) is a non-histone, chromatin-binding protein with an important role in chromatin remodeling [14]. HMGA1 binds to nuclear chromatin at AT-rich regions located in the minor grooves of DNA, where it bends the chromatin, allowing additional TFs to bind, thereby influencing gene regulation [14,15]. It is involved in DNA repair, cell cycle progression, cell proliferation, cell metabolism, and apoptosis [16,17,18]. Additionally, HMGA1 prevents embryonic stem cell differentiation by maintaining high levels of OCT4, NANOG, SOX2, and MYC [19]. Consequently, HMGA1 suppression decreased the expression of the pluripotency genes [20]. The HMGA1 protein is also found overexpressed in numerous aggressive human cancers [21,22,23,24]. It acts as an oncogene, and its high expression in cancer has been implicated in malignant transformation, tumor progression, and metastasis, while correlated with unfavorable clinical outcomes of the patients [23,25,26,27,28,29,30]. Accordingly, studies using tumor tissue specimens revealed that high HMGA1 expression is associated with a poor differentiation state and advanced disease or high-grade cancers [31,32,33]. Although the specific role and molecular mechanisms of HMGA1 in cancer have not been fully uncovered, studies have revealed that HMGA1 overexpression induced cancer cell proliferation, migration/invasion, and contributed to drug resistance in several tumors, including GC [16,17,18,25,34,35,36,37,38,39,40,41]. In GC, HMGA1 was also shown to stimulate epithelial–mesenchymal transition (EMT), thereby favoring a malignant progression [27]. It was also shown to participate in the regulation of aerobic glycolysis by regulating C-MYC expression [17]. However, the relevance of HMGA1 as a predictive biomarker with potential clinical applications has not been assessed in GC. Thus, the aim of this study was to investigate the clinical value of HMGA1 expression in a robust series of gastric adenocarcinoma cases with clinicopathological and treatment data. The importance of HMGA1 in predicting prognosis and response to therapy in GC patients was assessed by analyzing its expression in 323 cases from a consecutive, single hospital series. We observed that patients having tumors with high expression of HMGA1 benefit significantly more from chemotherapy compared to those with low expression. This observation can irrefutably contribute to a more efficient disease monitoring and treatment planning in GC patients.

## 2. Materials and Methods

### 2.1. Patients

Histological materials from 323 GC patients, mean age 67.68 ± 11.85, were collected from surgical specimens of consecutive cases of gastric adenocarcinoma surgically treated between January 2008 and December 2014 at Centro Hospitalar São João (CHSJ), Porto, Portugal. For all patients (*n* = 323), tumor tissue, clinicopathological and treatment data, as well as follow-up information were available. In addition to surgery, 121 patients out of 323 received platinum- and/or fluoropyrimidine-based chemotherapy, mostly as adjuvant treatments. Relevant clinical information on this series is provided in Mesquita et al. [42] and in the Table S1.

### 2.2. Tissue Microarrays and Immunohistochemistry

Cores from formalin-fixed, paraffin-embedded tumor tissue blocks from the surgical specimens were used to construct tissue microarrays (TMAs). After construction of the TMAs, they were sectioned with a microtome (Microm HM 335 E) at a thickness of 4 µm, and the expression of HMGA1 was evaluated by immunohistochemistry (IHC) staining using standard protocols described in Mesquita et al. [42]. Rabbit recombinant monoclonal HMGA1 primary antibody (anti-HMGA1, 1:5000 dilution, Abcam ab252930, Cambridge, UK) was incubated overnight at 4 °C in a humidified chamber. Samples were considered HMGA1-low when they were negative for HMGA1 or it was present in less than 20% of the malignant cells and considered HMGA1-high otherwise, in agreement between two observers.

### 2.3. Statistical Analysis

We aimed to evaluate the association between the expression status of HMGA1 and the clinicopathological features of the tumors, for which we applied different statistical tests. To compare using the patient’s age, we used Student’s *t*-test. For gender, tumor growth pattern, vascular invasion, perineural invasion, and treatment, we applied Fisher’s exact test (2-sides). For the Laurén classification and tumor node metastasis (TNM) staging, we used the chi-square test (χ^2^). We also aimed to investigate the association between HMGA1 expression status and risk of death or relapse. This was done using the Kaplan-Meier method in order to generate plots and survival curves for five-year overall survival (OS—time from operation to death from any cause) and disease-free survival (DFS—time from surgery to the first event of either locoregional recurrence or metastasis or death from the same cancer) that were compared using the log-rank test. To assess whether HMGA1 expression could predict response to chemotherapy, OS and DFS plots were generated according to the expression status of this protein and administration or not of chemotherapy. The Cox proportional hazard model was used to calculate univariate and multivariate hazard ratios and confidence intervals for death. The clinicopathological parameters included in the multivariate models were selected based on their individual clinical relevance. Patients with incomplete data were excluded from the analyses. Statistical analysis of the data was performed using the IBM SPSS Statistics version 24 (IBM Corporation, Armonk, NY, USA). Differences were considered statistically significant when *p* value < 0.05.

## 3. Results

### 3.1. Association of HMGA1 Expression with Clinicopathological Features in Gastric Carcinomas

**Figure 1:**
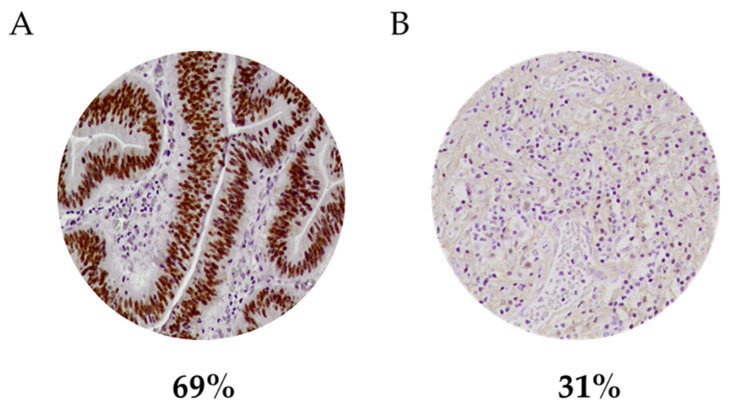
Representative immunostaining patterns for HMGA1 protein expression in two different GC cases and the respective percentages in the 323 gastric carcinomas evaluated (20× magnification). (**A**) GC case with high expression of HMGA1 in the nucleus (brown); 69% of gastric carcinomas express high levels of HMGA1 in the nucleus. (**B**) GC case with low HMGA1 expression; absence or low levels of HMGA1 were observed in 31% of gastric carcinoma cases. Tissues were counterstained with hematoxylin (purple).

HMGA1 expression was analyzed by IHC in 323 gastric carcinomas (Figure 1). Expression of HMGA1 was nuclear. High HMGA1 expression was observed in 223 (69%) gastric carcinoma cases (Figure 1A). HMGA1 was absent or low in 100 (31%) cases (Figure 1B). Clinicopathological features of the 323 GC cases and their association with HMGA1 expression are summarized in Table 1.

High levels of HMGA1 were significantly associated with tumors classified as intestinal (in 78% of the cases) or mixed (in 71% of the cases) according to the Laurén classification (*p* < 0.001), in opposition to tumors of the diffuse type (only in 44% of the cases) (Table 1). The other clinicopathological parameters, which included age at diagnosis, gender, growth pattern, TNM, and vascular and perineural invasion were not significantly associated with HMGA1 expression.

### 3.2. Evaluation of the Prognostic Significance of HMGA1 Expression in GC

**Figure 2:**
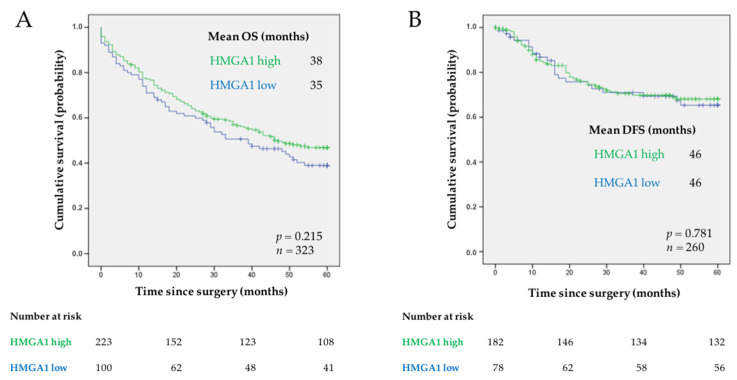
Kaplan-Meier curves showing the probability of (**A**) OS and (**B**) DFS in our series of patients with GC according to HMGA1 expression. Mean OS is shown for each subgroup of patients. The log-rank test was used to test for differences in survival between cases with high and low expression of HMGA1. *p* value is indicated. The number of patients at risk is specified for 0, 20, 40, and 60 months.

**Figure 3:**
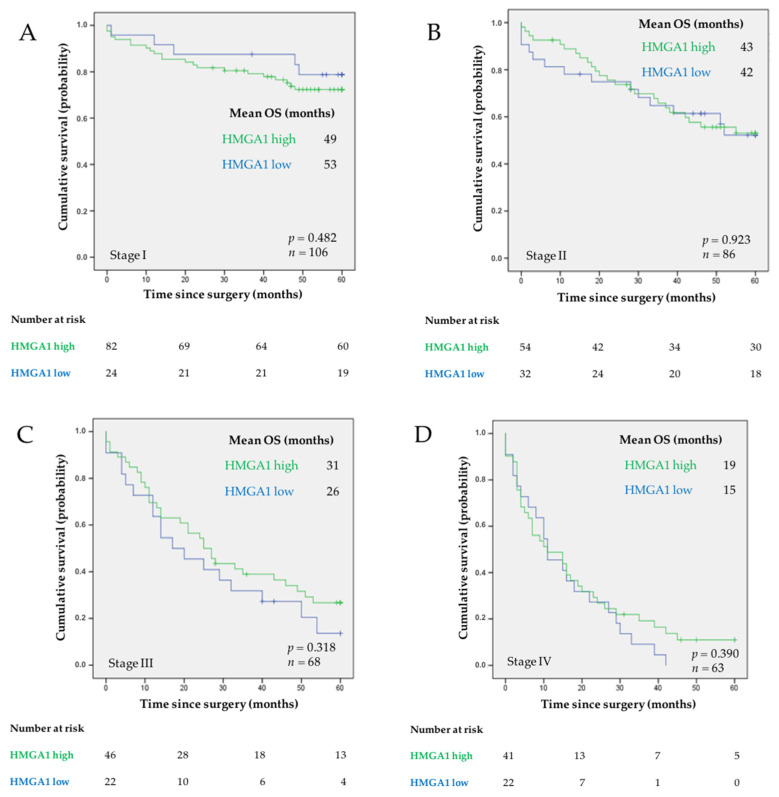
Kaplan-Meier curves showing the probability of OS according to HMGA1 expression in different stages of GC: (**A**) stage I, (**B**) stage II, (**C**) stage III, and (**D**) stage IV. Mean OS is shown for each subgroup of patients. The log-rank test was used to test for differences in survival between cases with high and low expression of HMGA1. *p* value is indicated. The number of patients at risk is specified for 0, 20, 40, and 60 months.

To assess the relevance of HMGA1 as a biomarker in our series of 323 patients diagnosed with GC, we started by evaluating if HMGA1 expression predicted patient OS or DFS using the Kaplan-Meier method. HMGA1 showed no significant relevance as a prognostic biomarker (Figure 2A,B) when considering all the patients or when patients were stratified by the TNM stage (Figure 3A–D).

### 3.3. Evaluation of the Predictive Value of HMGA1 for Response to Chemotherapy in GC

**Figure 4:**
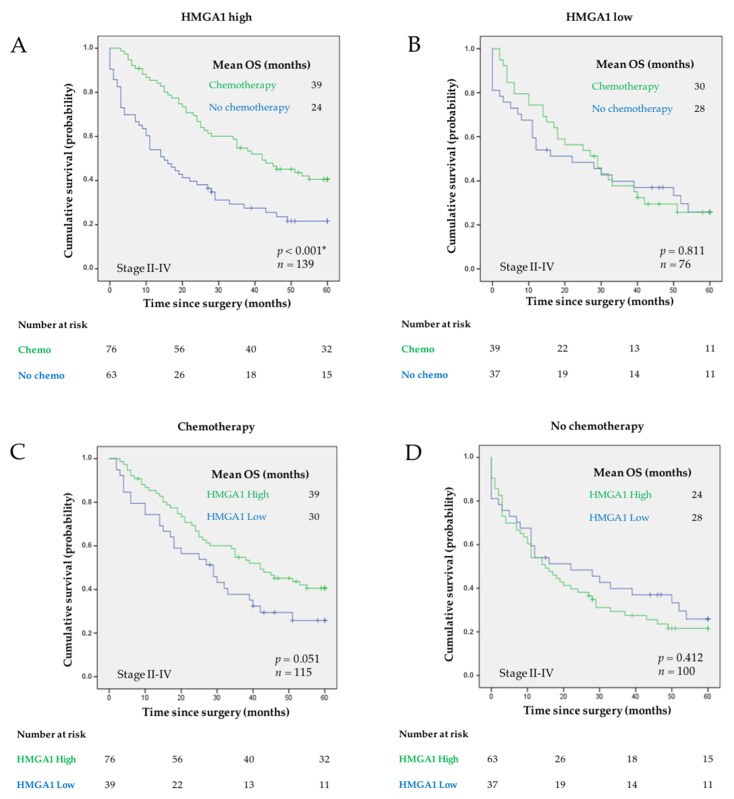
Kaplan-Meier curves showing the probability of OS according to treatment options in patients having tumors with: (**A**) high and (**B**) low expression of HMGA1, respectively. Alternatively, Kaplan-Meier curves showing the probability of OS according to levels of expression of HMGA1 in patients (**C**) treated or (**D**) not treated with chemotherapy. Mean OS is shown for each subgroup of patients. The log-rank test was used to test for differences in OS between treatment and absence of treatment (**A**,**B**) or between high and low levels of HMGA1 (**C**,**D**). *p* value is indicated. * comparisons with *p* < 0.05. The number of patients at risk is specified for 0, 20, 40, and 60 months.

Regarding treatment, 121 (37.5%) patients received chemotherapy, which improved OS [42]. The value of HMGA1 as a biomarker of response to chemotherapy was investigated. We observed a significantly better outcome in cases that had high levels of HMGA1 when they were treated with chemotherapy, comparing with those not treated (Figure 4A). The mean OS of patients with HMGA-high tumors treated with chemotherapy was 39 ± 2 months versus 24 ± 3 months in nontreated patients (*p* < 0.001), and indeed the combination of HMGA1-high expression and no treatment defined the group of patients with the worst outcome regarding OS. We did not observe a significant difference in cases with low levels of HMGA1, with or without chemotherapy (Figure 4B). Accordingly, in the universe of cases treated with chemotherapy, we saw a better outcome for the cases with high expression of HMGA1 (mean OS of 39 ± 2 months) compared to those with low expression (mean OS of 30 ± 3 months), although not significant (Figure 4C; *p* = 0.051). In the absence of chemotherapy, we did not observe a significant difference in OS between cases with different levels of HMGA1 expression (Figure 4D). The correlation between HMGA1 expression and adjuvant chemotherapy regarding DFS was also investigated (Figure S1), but we have not observed statistically significant differences.

**Figure 5:**
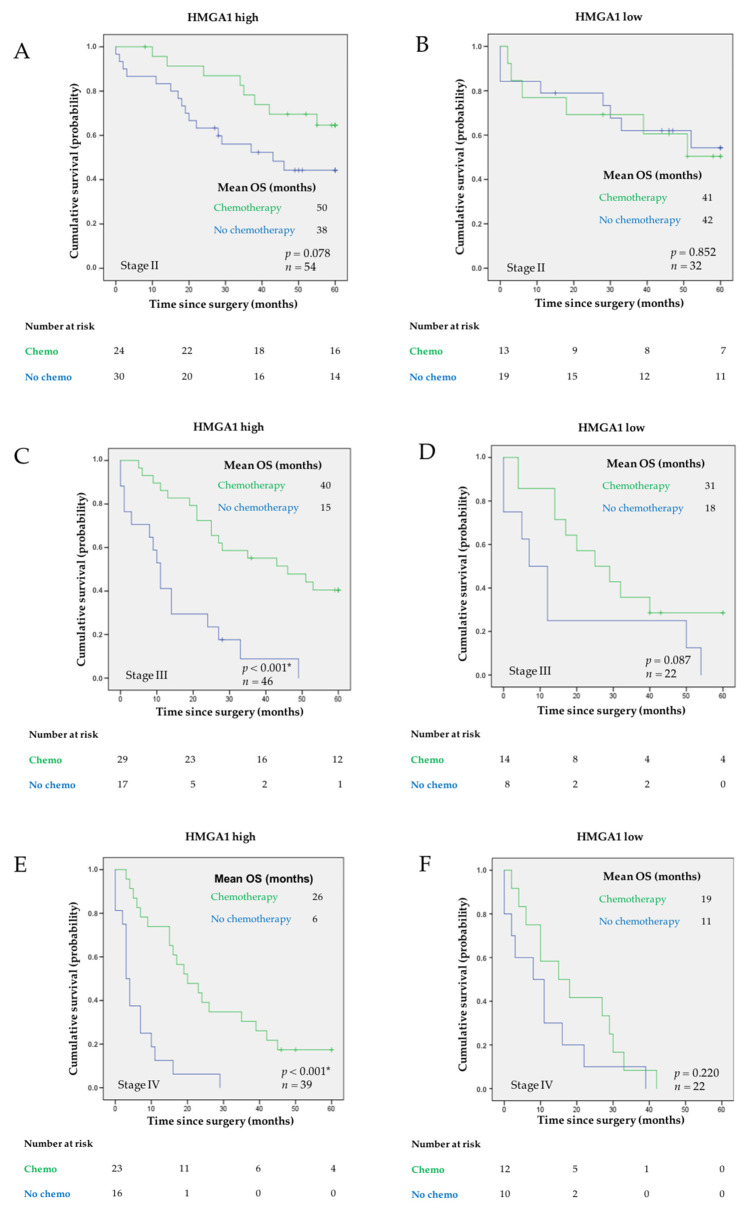
Kaplan-Meier curves showing the probability of OS according to treatment options in patients having tumors with: (**A**,**C**,**E**) high and (**B**,**D**,**F**) low expression of HMGA1, in patients diagnosed in: (**A**,**B**) stage II, (**C**,**D**) stage III, and (**E**,**F**) stage IV, respectively. Mean OS is shown for each subgroup of patients. The log-rank test was used to test for differences in survival between treatment and absence of treatment with chemotherapy. *p* value is indicated. * comparisons with *p* < 0.05. The number of patients at risk is specified for 0, 20, 40, and 60 months.

Independently analyzing the cases diagnosed in each TNM stage—II to IV—we observe a clear and significant benefit in treating the tumors diagnosed in stages III and IV that have high expression of HMGA1 (*p* < 0.001 for both stages), whereas the same was not true for those presenting low expression (Figure 5A–F). In stage III, the mean OS for patients having tumors with high levels of HMGA1 was 40 ± 4 months in patients treated with adjuvant chemotherapy in addition to surgery, compared to 15 ± 4 months in patients not treated. In stage IV, patients having tumors with high levels of HMGA1 that were treated with palliative chemotherapy had a mean OS of 26 ± 4 months while those not treated had a mean OS of 6 ± 2 months.

Furthermore, HMGA1 expression was independently associated with a lower risk of death in a Cox multivariate analysis restricted to patients diagnosed in stages II–IV treated with chemotherapy and that includes relevant clinicopathological factors such as Laurén classification, growth pattern, TNM staging, and vascular and perineural invasion as covariates (Table 2; HR = 0.53; 95% CI 0.30–0.95; *p* = 0.03).

## 4. Discussion

In the present study, we determined the value of nuclear HMGA1 expression in predicting GC’s prognosis and response to treatment, in addition to its association with clinicopathological features of the tumors. This assessment was performed using a series of 323 gastric carcinomas, the largest series of tumors used to date for this purpose and the most complete in terms of clinicopathological features, survival data, and treatment data. 

We found high levels of nuclear expression of HMGA1 in 69% of the tumors, which is comparable to those observed in all the reports published so far in the series of GC cases: 59% [28], 75% [43], and 74% [30]. An association between high expression of HMGA1 and Laurén intestinal and mixed-type tumors was observed in our series, and this agrees with previous reports of HMGA1 expression in gastric glands [18]. None of the other clinicopathological features were associated with HMGA1 expression. Accordingly, Nam et al. (2003) and Jun et al. (2015) reported no association at all between HMGA1 expression and clinicopathological features evaluated in a series of 62 and 110 GC cases, respectively, despite their analysis not including Laurén mixed-type tumors [30,43]. Contrarily, Yang et al. (2021) observed that increased HMGA1 expression was closely related with differentiation, lymph node metastasis, tumor size, and TNM stage, but this study was performed in a small series with only 51 GC samples [28].

To the best of our knowledge, our study is the first to identify HMGA1 expression as a useful marker to predict response to chemotherapy in GC. Our results unequivocally showed that patients with high levels of HMGA1 expression in the tumor had remarkably better OS rates when undergoing treatment with chemotherapy. This suggests that these patients can benefit more from this type of treatment than patients with low HMGA1 expression, where no significant difference was observed between treated and untreated patients. In the literature, there are several reports in cancer cell lines describing the role of HMGA1 expression in conferring resistance to antineoplastic drugs [44,45,46], including in GC [41]. These observations are in apparent contradiction with our results, if we consider only the association of HMGA1 with chemoresistance [47]. However, when we consider that HMGA1-high expression has been associated with features of higher malignancy in GC, such as increased proliferation, migration, metastasis, and EMT [18,27,28], the observation that HMGA1-tumors have better outcomes when subjected to more aggressive treatments is not surprising. Our results suggest that this parameter should be considered to determine which patients may benefit more from intensive chemotherapy management in an attempt to perform a more personalized medicine. This conclusion has a great impact because it advises for the treatment with chemotherapy of the 65% of GC patients with high HMGA1 expression diagnosed in stages II–IV. Considering only the cases with high expression of HMGA1 detected in stages III and IV, where the significant and most striking differences were obtained in mean OS values, our data supports the selection of 40% of the GC patients diagnosed in all tumor stages, except stage I, for treatment. Besides this role we describe for HMGA1 in predicting chemotherapy response, the role described for HMGA1 as a glycolysis regulator in gastric cancer suggests that the HMGA1-c-Myc-glycolysis axis may also be used as a new target in cancer therapy in combination with commonly used chemotherapeutic agents [17].

High expression of HMGA1 has already been correlated with worse survival in GC patients but only in studies that used series with a relatively small number of cases [28,43] or TCGA/GEPIA datasets [17,27]. High expression of another member of the family, HMGA2, has also been reported as predicting bad prognoses in two series of Asian GC patients [43,48]. However, in our series, we did not observe a prognostic value for HMGA1. This somewhat intriguing result can only be explained by our observation that chemotherapy modifies the course of the disease for patients having high expression of HMGA1 in the tumor, increasing the OS of these cases. In fact, when we considered only the patients treated with chemotherapy, we observed that HMGA1 expression lowers the risk of death to one half, independently from relevant clinicopathological factors.

## 5. Conclusions

Additional studies are needed, but our observation that GC patients with high levels of HMGA1 expression in the tumor benefit more from being treated with chemotherapy, should advise the selection of these patients for treatment, with expected improvements of their overall survival.

## Figures and Tables

**Table 1:** Clinicopathological association with HMGA1 expression in 323 patients diagnosed with GC.

	All Cases		HMGA1 High		HMGA1 Low		*p*
	*n*	%	*n*	%	*n*	%	
Patients	323		223	69.0	100	31.0	
Age							
Mean ± SD	67.68 ± 11.85		68.26 ± 11.61		66.39 ± 12.32		0.19
Range	32–95		33–95		32–87		
Gender							
Female	140	43.3	100	71.4	40	28.6	0.47
Male	183	56.7	123	67.2	60	32.8	
Laurén Classification							
Intestinal	151	46.8	117	77.5	34	22.5	<0.001 *
Diffuse	41	12.7	18	43.9	23	56.1	
Mixed	85	26.3	60	70.6	25	29.4	
Unclassified	46	14.2					
Growth Pattern							
Expansive	58	18.0	38	65.5	20	34.5	0.53
Infiltrative	252	78.0	176	69.8	76	30.2	
Unclassified	13	4.0					
TNM							
I	106	32.8	82	77.4	24	22.6	0.14
II	86	26.6	54	62.8	32	37.2	
III	68	21.1	46	67.6	22	32.4	
IV	63	19.5	41	65.1	22	34.9	
Vascular Invasion							
No	134	41.5	93	69.4	41	30.6	1.00
Yes	186	57.6	128	68.8	58	31.2	
ND	3	0.9					
Perineural Invasion							
No	167	51.7	120	71.9	47	28.1	0.28
Yes	156	48.3	103	66.0	53	34.0	
Chemotherapy treatment							
No	200	61.9	141	70.5	59	29.5	0.46
Yes	121	37.5	80	66.1	41	33.9	
ND	2	0.6					

Notes: *p* values (statistical significance threshold < 0.05) were obtained using Student’s *t* test for the continuous variable, Fisher’s exact test (2-sided) and Chi-square (χ^2^) test for categorical variables; * comparisons with *p* < 0.05. ND = not determined; SD = standard deviation.

**Table 2:** Overall survival univariate and multivariate Cox regression analysis in gastric cancer patients diagnosed in stages II–IV and treated with chemotherapy.

	Number of Events	Univariate Analysis			Multivariate Analysis		
		HR	95% CI	*p*-Value	HR	95% CI	*p*-Value
HMGA1							
Low	28	1			1		
High	44	0.63	0.39–1.01	0.06	0.53	0.30–0.95	0.03 *
Laurén Classification							
Intestinal	23	1			1		
Diffuse	15	1.75	0.91–3.36	0.09	1.33	0.61–2.89	0.47
Mixed	23	1.51	0.84–2.27	0.17	1.06	0.56–1.98	0.87
Growth pattern							
Infiltrative	65	1			1		
Expansive	5	0.74	0.30–1.83	0.51	0.82	0.31–2.16	0.68
TNM							
II	14	1			1		
III	27	2.04	1.07–3.89	0.03 *	2.03	0.96–4.29	0.06
IV	32	4.40	2.31–8.38	<0.001 *	4.41	2.08–9.34	<0.01 *
Vascular Invasion							
No	20	1			1		
Yes	52	1.34	0.80–2.24	0.27	1.81	0.97–3.38	0.06
Perineural Invasion							
No	20	1			1		
Yes	52	1.65	0.99–2.78	0.06	1.09	0.58–2.03	0.80

Notes: *p* values (statistical significance threshold < 0.05) were obtained using univariate and multivariate Cox proportional hazards regression analysis (Wald); * comparisons with *p* < 0.05. HR = Hazard-ratio; CI = Confidence interval.

## Data Availability

The datasets used and/or analyzed during the current study are available from the corresponding authors on reasonable request.

## Supplementary Materials

The following are available online at https://www.mdpi.com/article/10.3390/curroncol29010005/s1, Figure S1: Kaplan-Meier curves showing the probability of DFS according to treatment options, Table S1: Chemotherapy regimens used in the 121 treated GC patients, namely in cases having either high or low expression of HMGA1.
