# Supplementary material for: HMGA1 Has Predictive Value in Response to Chemotherapy in Gastric Cancer
Source: Curr Oncol. 2021 Dec 23;29(1):56–67. doi: 10.3390/curroncol29010005 (PMC8774981; doi:10.3390/curroncol29010005)
Supplement: Supplementary file 1 [file curroncol-29-00005-s001.zip › curroncol-1491628-supplementary.pdf]

Current Oncology

MDPI

Supplementary Material

HMGA1 has Predictive Value in Response to Chemotherapy in Gastric Cancer

Diana Pádua, Débora Filipa Pinto, Paula Figueira, Carlos Filipe Pereira, Raquel Almeida and Patrícia Mesquita

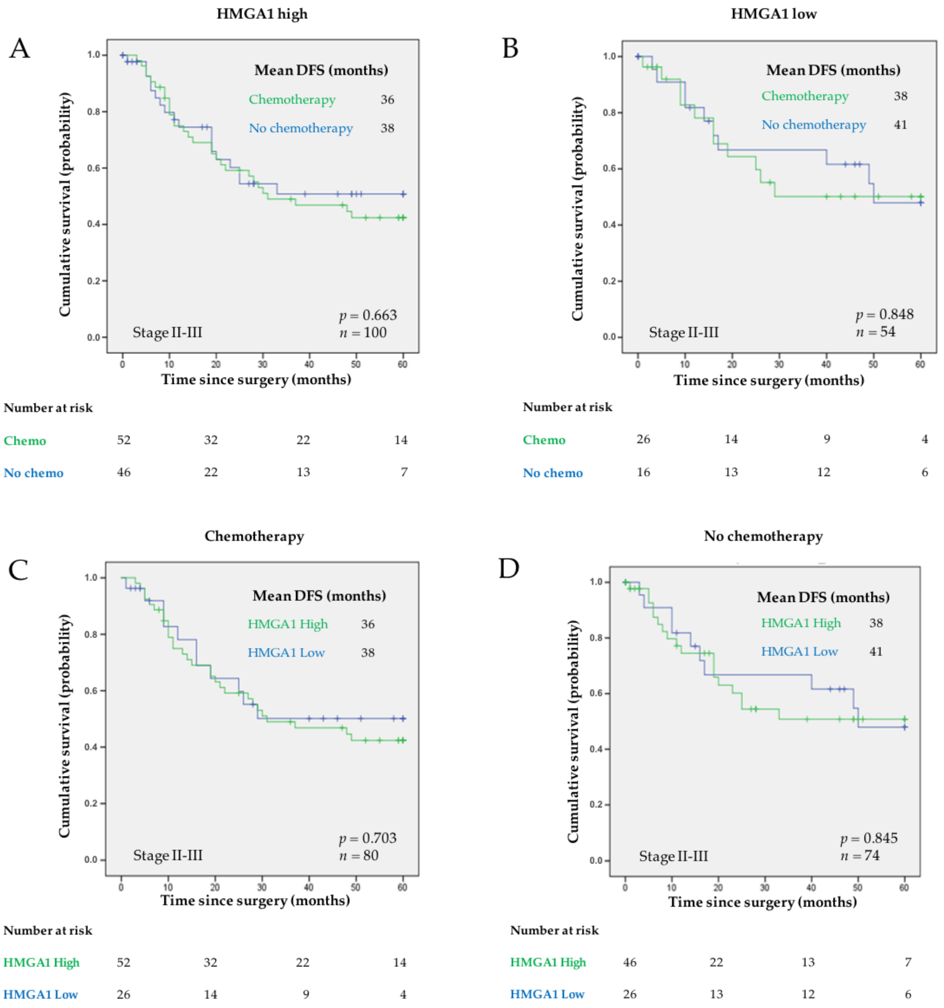

Figure S1. Kaplan-Meier curves showing the probability of DFS according to treatment options in patients having tumors with: (A) high and (B) low expression of HMGA1, respectively. Alternatively, Kaplan-Meier curves showing the probability of DFS according to levels of expression of HMGA1 in patients (C) treated or (D) not treated with adjuvant chemotherapy. Mean DFS is shown for each subgroup of patients. The log-rank test was used to test for differences in DFS between treatment and absence of treatment (A and B) or between high and low levels of HMGA1 (C and D). p value is indicated. The number of patients at risk is specified for 0, 20, 40 and 60 months.

Table S1. Chemotherapy regimens used in the 121 treated GC patients, namely in cases having either high or low expression of HMGA1.

**Chemotherapy Regimens**			**HMGA1 high**		**HMGA1 low**	
**Drugs**	***n***	**%**	***n***	**%**	***n***	**%**
Carboplatin and Docetaxel	1	0,9	1	1,3	0	0,0
Carboplatin, 5-Fluorouracil and Taxotere	3	2,6	3	4,0	0	0,0
Cisplatin and 5-Fluorouracil (CF)	9	7,8	7	9,2	2	5,1
Cisplatin, 5-Fluorouracil and Etoposide	2	1,7	1	1,3	1	2,6
Cisplatin, 5-Fluorouracil and Taxotere	26	21,8	14	17,1	12	30,7
Cisplatin, 5-Fluorouracil and Docetaxel (modified DCF)	5	4,3	4	5,3	1	2,6
Cisplatin, Epirubicin and continuous 5-Fluorouracil (ECF)	2	1,7	0	0,0	2	5,1
Cisplatin and Capecitabine (XP)	25	20,9	16	19,7	9	23,1
Cisplatin, Capecitabine and Taxotere	1	0,9	1	1,3	0	0,0
Capecitabine (monotherapy)	6	5,2	4	5,3	2	5,1
Capecitabine, Epirubicin and Oxaliplatin (EOX)	16	11,3	12	13,2	4	7,7
Capecitabine and Oxaliplatin (XELOX)	16	13,9	11	14,5	5	12,9
Capecitabine and Irinotecan	1	0,9	1	1,3	0	0,0
Leucovorin calcium (folinic acid), 5-Fluorouracil and Irinotecan (FOLFIRI)	2	1,7	2	2,6	0	0,0
Leucovorin calcium and 5-Fluorouracil	1	0,9	1	1,3	0	0,0
Leucovorin calcium (folinic acid), 5-Fluorouracil and Oxaliplatin (FOLFOX)	1	0,9	1	1,3	0	0,0
No information	4	2,6	1	1,3	3	5,1
Total	121	100	80	100	41	100
